# The Role of Glucocorticoids in the Treatment of Multisystem Inflammatory Syndrome (MIS-C)—Data from POLISH MIS-C Registry

**DOI:** 10.3390/children9020178

**Published:** 2022-02-01

**Authors:** Ewelina Gowin, Kacper Toczyłowski, Artur Sulik, Jacek Wysocki, Danuta Januszkiewicz-Lewandowska

**Affiliations:** 1 Health Promotion Department, Poznan University of Medical Sciences, Fredry 10, 61-701 Poznan, Poland; jwysocki@ump.edu.pl; 2 Department of Pediatric Infectious Diseases, Medical University of Bialystok, Waszyngtona 17, 15-274 Bialystok, Poland; kacper.toczylowski@umb.edu.pl (K.T.); artur.sulik@umb.edu.pl (A.S.); 3 Department of Pediatric Oncology, Hematology and Transplantology, Poznan University of Medical Sciences, Szpitalna 27/33, 60-572 Poznan, Poland; danuta.januszkiewicz@ump.edu.pl

**Keywords:** multisystem inflammatory syndrome (MIS-C), fever, Kawasaki disease

## Abstract

Background: Multisystem inflammatory syndrome (MIS-C) is a condition related to COVID-19. It’s most significant feature is cardiac involvement. Methods: We have analyzed data from 42 hospitals in the Polish MIS-C Registry. To compare the effect of GCS on fever, we formed two groups: the first treated with IVIG and the second treated with IVIG+GCS. Results: There were 111 boys and 56 girls; the mean age was 8.57 years. All the patients were treated with IVIG: 76 patients with IVIG only, and 91 patients with IVIG+GCS. There were no statistically significant differences between the groups regarding age, gender, BMI, or inflammatory markers. Methylprednisolone was the most common drug (80%). Echocardiographic abnormalities on admission were more prevalent in the IVIG+GCS group. Mean time from IVIG infusion to subsidence of fever was 1.1 days, and 1.5 for those in the IVIG+GCS group. Conclusions: GCS are commonly used in the treatment of MIS-C patients in Poland. Various GCS regimens are used, from a single dose to a month-long therapy. Children with lower lymphocyte levels and cardiac abnormalities on an echocardiographic examination performed on admission were more likely to receive GCS+IVIG. The effect of GCS is difficult to access as patients were not randomly assigned to receive the treatment.

## 1. Introduction

Multisystem inflammatory syndrome (MIS-C) is a condition related to COVID-19. The first cases of MIS-C reported in EU/EEA countries and the UK were in spring 2020 [1,2,3,4].

Lucio Verdoni et al., in an article published in Lancet, described the first outbreak of Kawasaki disease (KD) in Italy [2]. Epidemiological data reveal that MIS-C occurred 3–8 weeks after exposure to SARS-CoV-2. This suggests that MIS-C is a post-infectious inflammatory process likely driven by dysregulation of the adaptive response. The clinical spectrum of MIS-C may resemble KD. However, it has become evident that shock, gastrointestinal symptoms, and coagulopathy, which are rarely seen in classic KD, are prominent features of this unique syndrome [5,6]. Children with MIS-C required aggressive management strategies. Intensive care admissions were significantly more common for MIS-C than for KD patients [5].

The first attempts at treatment were based on experiences with patients with KD. Based on current guidelines, the necessary treatment for KD is intravenous immunoglobulin (IVIG) (2 g/kg) used with acetylsalicylic acid (ASA) [7,8,9]. Additional treatment is based on glucocorticoids (GCS) and newer anti-inflammatory drugs. In recent years, IVIG plus GCS combination therapy has been attracting attention both as an additional treatment for IVIG-resistant KD patients and as an initial treatment [10,11]. Newburger found that primary therapy with pulsed intravenous methylprednisolone, administered as a single dose of 30 mg per kilogram before conventional treatment with IVIG (2 g per kilogram), did not improve coronary-artery outcomes at week one or week five after study enrollment [12]. In Japan, the RAISE study was a 74-hospital, randomized study of intravenous prednisolone 2 mg/kg/day for five days followed by a tapering oral regimen. High-risk patients had been identified through a scoring system and randomized to receive either prednisone or placebo with standard IVIG therapy for four to five weeks until the normalization of C-reactive protein (CRP) [13]. The results show that patients in the steroid arm had less fever and a more significant coronary artery Z score reduction. According to a Post-RAISE study, a primary IVIG and prednisolone combination therapy might prevent coronary artery abnormalities [14]. Three studies assessed single-dose methylprednisolone (30 mg/kg/day) for children predicted to be IVIG-resistant and found that the steroid group had better treatment outcomes.

Meta-analysis of 16 comparative studies involving KD patients demonstrated that the early addition of GCS to conventional IVIG therapy is associated with a reduced risk of cardiac complications. The best effect was observed when corticosteroids were used as primary therapy [15]. The RISE study proved the beneficial effect in patients with a high risk of developing cardiac complications [13]. GCS are widely used in treating many different inflammatory conditions, but their role in the treatment of KD has been discussed for many years [14,16].

In this study, we wanted to analyze the role of GCS in the treatment of Polish MIS-C patients. We investigated indications for the GCS treatment and the conventional regimen. The effect of GCS was checked based on the duration of fever after the introduction of the treatment.

## 2. Material and Methods

We analyzed data from the Polish MIS-C Registry [17]. Anonymized patient data from 42 pediatric hospitals from all over the country were extracted from electronic and paper records and collected through an online form developed for that purpose. Demographic data, clinical characteristics, laboratory parameters, cardiovascular evaluation results, treatment, and outcome data were collected. Vital signs and laboratory parameters were obtained at admission and their respective peaks. Here we report the data covering 4 March 2020 (when the first COVID-19 case was confirmed in Poland) and 20 April 2021.

**Figure 1:**
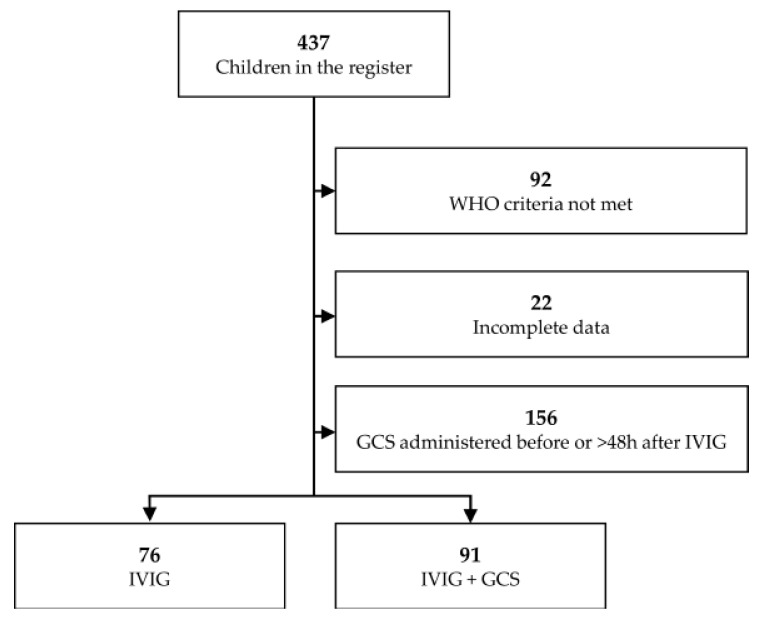
Study flow diagram illustrating the process of selecting patients for inclusion in the study; abbreviations: GCS—glucocorticoids, IVIG—intravenous immunoglobulins.

MIS-C was diagnosed based on the published guidelines from the Royal College of Pediatrics and Child Health, World Health Organization (WHO), and Centers for Disease Control and Prevention (CDC) [7,8,9]. The principal investigators adjudicated cases at each site and the central coordinating center. The case definition of MIS-C included six criteria: serious illness leading to hospitalization, an age of fewer than 21 years, fever (body temperature >38.0 °C) or report of subjective fever lasting at least 24 h, laboratory evidence of inflammation, multisystem organ involvement (i.e., involving at least two organ systems), and laboratory-confirmed SARS-CoV-2 infection (positive SARS-CoV-2 real-time reverse-transcriptase–polymerase-chain-reaction (RT-PCR) or antibody test during hospitalization) or an epidemiological link to a person with suspected or confirmed COVID-19 within four weeks before the onset of MIS-C symptoms. Owing to a lack of SARS-CoV-2 testing in the spring of 2020, cases reported between 15 March and 31 May as suspected or confirmed MIS-C cases that were epidemiologically linked to a COVID-19 exposure were included without the requirement of a positive SARS-CoV-2 test; positive testing was required after 31 May. In the registry, there were 437 patients. We excluded patients with incomplete GCS/IVIG treatment data or those who did not fill the WHO criteria (Figure 1).

To compare the effect of GCS on fever, we formed two groups: patients treated with IVIG only and a second group of patients who received GCS at the same time as IVIG (within 48 h of starting IVIG)— the study flow of which is presented in Figure 1. We excluded from the analysis children whose fever subsided before introduction of the treatment or who received steroids more than two days after IVIG infusion. Demographic characteristics, duration of clinical symptoms, and inflammatory markers were compared across the groups. Ethical approval was obtained from the Bioethics Committee at Wroclaw Medical University (CWN UMW BW: 39/2020).

The summary statistics for continuous variables are presented as a median with interquartile range (IQR), categorical variables are presented as frequencies. Differences between groups were analyzed by the Mann–Whitney U test or by the chi-squared test. The continuous variables were compared using the Mann–Whitney U test, categorical variables between groups using χ^2^ tests.

## 3. Results

Among the 437 pediatric patients reported to the Polish Registry with suspected MIS-C, 345 fulfilled WHO criteria for MIS-C associated with SARS-CoV-2 infection. All cases occurred between June 2020 and April 2021.

In the study group, there were 111 boys and 56 girls. All patients were of European white ethnicity; mean age was 8.57 years (range four months–17 years), 43 patients (25.7%) were younger than five years, while 84 patients (50.3%) were in the age group 5–12 years.

No patient had a personal or family history of KD. Details are presented in Table 1.

All the patients were treated with IVIG (1–2 g/kg to max 100 g given over two days): 76 patients were treated only with IVIG, 91 patients received combined treatment IVIG and GCS.

There were no patients treated with GCS without IVIG. Methylprednisolone (0.8–1 mg/kg b.i.d or pulses) was the most common drug (73/91-80% of patients treated with GCS). Dexamethasone (0.15 mg/kg/dose to max 6 mg once daily) was used in 17 patients (18.7% of patients treated with GCS). Oral prednisolone (1 mg/kg to max 60 mg) as the only steroid treatment was administered in one patient. Methylprednisolone pulses (30 mg/kg) were used in 54 patients (59.3% of patients treated with GCS) (only pulses in 3). In 51 patients, pulses were followed by decreasing doses of oral prednisolone. No children treated with extracorporeal membrane oxygenation (ECMO) or with renal replacement therapy. No deaths occurred in the study population; 6% of patients were treated at the intensive care unit, 94% had complete recovery at discharge. None of the patients required additional anti-inflammatory treatment other than IVIG/IVIG+GCS.

The effect of GCS on the duration of fever was compared in both groups—patients treated only with IVIG and patients who received GCS at the same time as IVIG. There were no statistically significant differences between groups regarding age, gender, or BMI. Inflammatory markers were similar in both groups. Children treated with combined treatment had lower mean levels of lymphocytes and higher activity of alanine aminotransferase (ALT). Increased ALT activity (≥25IU/l) was detected in 30.26% (23/76) IVIG treated patients and in 51.64% (47/91) of patients treated with IVIG+GCS.

Serum sodium level below 133 mmol/l was detected in 21.05% (16/76) of patients from the IVIG treated group and 35.16% (32/91) of patients from the IVIG+GCS treated group, platelet levels lower than 150,000/µL were detected in 19.74% (15/76) of patients in the IVIG group and 26.37% (24/91) of patients in the IVIG+GCS treated group.

Echocardiographic abnormalities on admission were more prevalent in the group treated with IVIG+GCS.

**Figure 2:**
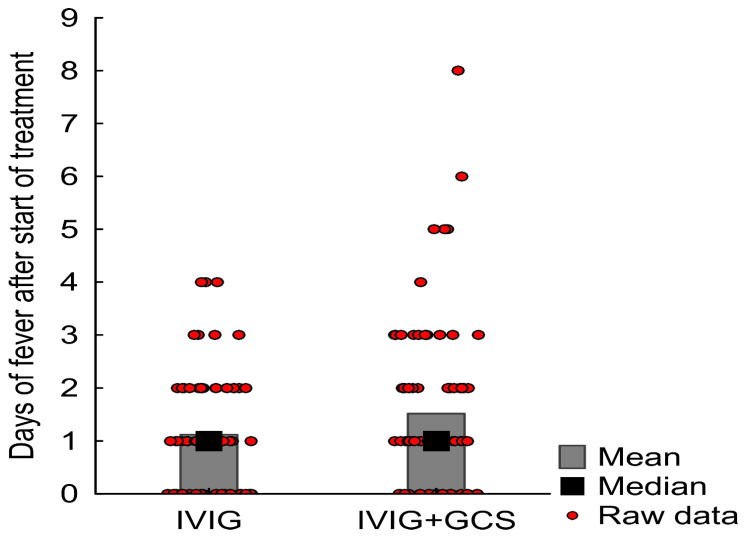
The effect of GCS on the duration of fever. GCS—glucocorticoids, IVIG—intravenous immunoglobulins.

Mean time from IVIG infusion to subsidence of fever was 1.1 days (SD 1.1, median value one day, IQR 0-2), and 1.5 days in the group treated with IVIG+GCS (SD 1.4, median value one day, IQR 1-2) (the difference is not of statistical significance; *p* = 0.08). Results are presented in Figure 2.

## 4. Conclusions

GCS are commonly used in the treatment of MIS-C patients in Poland.

Various GCS regimens are used, from a single dose to a month-long therapy.

Children with lower lymphocyte levels and cardiac abnormalities on an echocardiographic examination performed on admission were more likely to receive GCS+IVIG.

The effect of GCS is difficult to assess as patients were not randomly assigned to receive the treatment.

## 5. Discussion

Our analysis shows that GCS are commonly used in patients with MIS-C. Different regiments are used from small doses to pulses, one dose to month-long therapy. MIS-C treatment is based on guidelines dedicated to KD treatment [7,8,9].

GCS have anti-inflammatory and immunosuppressive effects. Prednisone, prednisolone, and methylprednisolone have intermediate plasma half-lives, whereas betamethasone and dexamethasone are long-acting analogs. Acute flares of inflammatory diseases are treated with pulse IV steroids. Orally administered GCS are rapidly absorbed and can be used for prolonged treatment. The effects of GCS are dose dependent. For KD, two regimens are commonly used. The first of these is as follows; methylprednisolone 0.8 mg/kg BD i.v. for 5–7 days or until CRP normalizes, then convert to oral prednisone/prednisolone 2 mg/kg/day and wean off over the subsequent 2–3 weeks. The second regimen requires methylprednisolone 10–30 mg/kg (up to a maximum of 1 g/day) once daily for three days followed by oral prednisone/prednisolone 2 mg/kg per day until day seven or until CRP normalizes, then wean over next 2–3 weeks [13].

Most of our patients were treated with doses resembling regimen one. Pulses of high doses of GCS are used for life-threatening conditions in rheumatology. The potential acute toxicities of pulse doses are hypertension or hypotension, tachycardia, bradycardia, cardiac arrhythmia secondary to potassium depletion, blurring of vision, hyperglycemia with or without ketosis, flushing, sweating, metallic taste in the mouth, acute psychosis, behavioral changes, convulsions, and anaphylaxis. These side effects can be dangerous to patients. No serious adverse effects were recorded during the study period.

Steroid pulses were used in many of our patients, but only in three from the group treated with only GCS. The idea of using pulses is to shorten the exposure to GCS. This was not the case in our patients, as most received treatment for longer than seven days. therefore, the usefulness of steroid pulse therapy should be discussed.

According to the American College of Rheumatology, high-dose, IV pulse GCS (10–30 mg/kg/day) may be considered in MIS-C patients who do not respond to IVIG and low-to-moderate-dose GCS, or if a patient requires high-dose inotropes and/or vasopressors [18]. In our study, children were treated with different types and doses of GCS, usually longer than seven days. Each time it was a decision of an individual clinician. Cases of MIS-C appeared in Poland later than in other European countries (autumn 2020). During the first wave in spring 2020, COVID-19 cases were mainly detected in long-term healthcare facilities for the elderly. Polish doctors were aware of how severe this disease can be, explaining the widespread use of a combined treatment.

The pathophysiology of MIS-C is not fully understood. The inflammatory response in MIS-C is similar to the immune response to acute COVID-19. Dexamethasone in a dose of 6 mg once daily for ten days turns out to be effective in treating adults with COVID-19 [19]. In our group, less than 20% of patients were treated with dexamethasone. According to Italian guidelines, preliminary data on the use of dexamethasone 10 mg/m^2^ should be used in secondary hemophagocytic lymphohistiocytosis or for central nervous system involvement [20].

Most of the recent studies comparing treatment with IVIG and methylprednisolone vs. IVIG alone have given positive results. In the French study among children with MIS-C, treatment with IVIG and methylprednisolone vs. IVIG alone was associated with a more favorable fever course. Combination therapy was also associated with less severe acute complications, including acute left ventricular dysfunction and hemodynamic support requirement [21]. The Overcoming COVID consortium (consisting of 58 U.S. hospitals) determined that treatment with IVIG plus GCS was associated with a lower risk of cardiovascular dysfunction and the initiation of vasopressors and adjunctive therapy than treatment with IVIG alone [22]. In contrast, the Best Available Treatment Study (BATS), performed in 32 countries, found statistically significant differences in primary outcomes (i.e., inotropic support, mechanical ventilation, or death) in children who had received combination IVIG and GCS together, or either drug in isolation [23]. The risk of escalation of immunomodulatory treatment in patients who received IVIG plus GCS was significantly lower than the risk in patients who received IVIG alone. This finding was in line with the results of the U.S. study [22,24]. None of our patients required additional immunomodulatory treatment. The overall effect of GCS in MIS-C is a shorter duration of fever—a vital clinical sign of inflammation [21,22]. The same effect of GCS was observed in KD [13,25].

We found no evidence that recovery from MIS-C or fever subsidence differed after primary treatment with IVIG alone or IVIG plus GCS. The baseline differences in these groups can cause this lack of steroid effect. The decision on starting GCS was made by clinicians based on the patient’s presentation. Children with more severe manifestations probably received combined treatment. Lymphocytopenia is a common finding in patients with MIS-C [17,25,26]. Patients with lower mean lymphocyte levels or cardiac abnormalities detected on echocardiography were more likely to receive combined treatment in our group.

It is difficult to conclude the usefulness of GCS, while data from randomized studies are still insufficient. With the widespread occurrence of MIS-C, there is a need for affordable treatment.

Anti-inflammatory drugs other than GCS are not commonly used in children—the majority of pediatricians are not accustomed to ordering them [25]. As the disease is acute, children with MIS-C are treated in general hospitals, not only in rheumatology departments. GCS are widely available and can be administered even in district hospitals.

The other reason for looking toward IVIG-sparing treatment is the risk of shortages of IVIG [27]. The median age of MIS-C patients in our group was 8.6 years. This is in accordance with the most extensive reported series, and older age is one thing that distinguishes KD from MIS-C [1,2,3,4,26]. As obesity is a current problem in pediatrics, almost 20% of our patients were obese or overweight. Therefore, they were heavier than previous patients with KD, and thus the demand for IVIG is high. The upper limit of given IVIG is 100 g, which still generates vast costs. IVIG are not standard drugs—their composition varies over time. IVIG prepared from donations in 2021 may have anti-SARS-CoV-2 antibodies, but we do not know the effect of these antibodies on the inflammation.

Our study has certain limitations. First, it was not a randomized trial. The decision about the treatment was individualized. More severely ill patients could have been treated more aggressively, and such an approach would have minimized the beneficial effect in the group that received IVIG+GCS. Second, there was variation in the dosage and routes of steroid treatment used, and the study design did not allow for comparing regimens. Patients were presented to the hospital at different times in the natural course of the disease. Disease severity was considerably higher in the US and French studies compared with our group [21,23].

Our study builds on earlier studies of MIS-C, giving insight into some differences in MIS-C in different parts of the world. The three most significant observational studies varied in disease severity and inclusion criteria, treatments compared, and outcomes measured, but all found clinical benefit with IVIG+GCS compared with IVIG alone [20,21,23]. Combination therapy was associated with a lower risk of persistent or recurrent fevers, reduced need for adjunctive immunomodulatory therapy or hemodynamic support. Our study demonstrated GCS+IVIG is the treatment most widely used in Poland and may be beneficial for MIS-C patients.

## Figures and Tables

**Table 1:** Demographic characteristics of the study group and comparison between the group treated with IVIG and the group treated with IVIG+GCS.

	IVIG *n* = 76	IVIG+GCS *n* = 91	*p*
Sex m/f (%m)	46/30 (61%)	65/26 (71%)	0.14
Age, years	8.7 (4.6–11.3)	9.4 (5.2–12.5)	0.15
Body mass index z-score	−0.03 (−0.79–0.70)	0.41 (−0.65–1.16)	0.06
Obesity	3%	7%	0.27
Fever before hospitalization, days	4 (3–6)	4 (2–4)	0.17
Skin rash, (%)	81%	89%	0.18
Conjunctivitis, (%)	79%	86%	0.27
Oral inflammation, (%)	62%	68%	0.42
Erythema of hands or feet, (%)	51%	60%	0.28
Gastrointestinal symptoms, (%)	92%	96%	0.33
Echocardiographic abnormalities, (%)	22%	50%	<0.001
CXR or CT abnormalities, (%)	16%	29%	0.06
Reduced left ventricle contractility on admission, %	9/70, 13%	30/84, 36%	0.001
Coronary artery dilatation on admission, %	3/70, 4%	9/84, 11%	0.14
Pericardial effusion on admission, %	5/70, 7%	6/84, 7%	1.0
Treated at PICU, (%)	1%	10%	0.02
Oxygen supplementation, *n* (%)	12%	28%	0.02
Complete recovery at discharge	98%	92%	0.09
CRP, mg/L	140.9 (88.4–187)	132.1 (69–185.2)	0.5
Lactate, mmoL/L	1.9 (1.2–2.4)	2.5 (1.8–3.2)	0.05
Hb on admission, g/dL	11.5 (10.3–12.7)	12.0 (11.1–13.4)	0.03
Anaemia on admission, %	48%	33%	0.056
WBC, ×10^3^/µL	10.6 (7.3–14)	8.3 (6.2–12.3)	0.03
Neutrophils, ×10^3^/µL	8 (5.7–11)	7 (4.7–10.5)	0.28
Lymphocytes, ×10^3^/µL	1.5 (1–2)	0.9 (0.6–1.4)	<0.001
PLT, ×10^3^/µL	196.5 (148–304)	159.5 (112–227)	0.002
D-dimers, mg/L	2.2 (1.4–3)	3.4 (1.5–5.6)	0.007
INR	1.2 (1.1–1.3)	1.3 (1.2–1.4)	0.001
APTT, s	34 (31.5–38.6)	36.9 (34.3–40.5)	0.03
ALT, U/L	19.5 (13–29.5)	27 (17–48)	<0.001
Bilirubin, mg/dL	0.4 (0.3–0.6)	0.6 (0.4–1.1)	0.05
Sodium, mmoL/L	135 (133–138)	134 (131–136)	0.007
NT pro-BNP, pg/mL	1581 (718–5791)	3966 (793–11416)	0.14
Creatinine, mg/dL	0.4 (0.3–0.6)	0.6 (0.4–0.7)	0.003
CK, U/L	40 (19–58)	59.5 (39–126)	0.008

ALT—alanine aminotransferase, BMI—body mass index, CRP—C-reactive protein, CK—creatinine kinase, CT—computed tomography, CXR—chest X-ray, GCS—glucocorticoids, INR—international normalized ration, IVIG—intravenous immunoglobulins, NT-pro-BNP—N-terminal pro b-type natriuretic peptide, PICU—pediatric intensive care unit, PLT—platelets, WBC—white blood cells.

## Data Availability

Data supporting reported results can be found in Polish MIS-C Registry.

## Abbreviations

ALT	alanine aminotransferase
ASA	acetylsalicylic acid
BMI	body mass index
CDC	Center for Disease Control and Prevention
CRP	C-reactive protein
CK	creatinine kinase
CT	computed tomography
CXR	chest X-ray
GCS	glucocorticoids
INR	international normalized ration
IQR	interquartile range
IVIG	intravenous immunoglobulins
KD	Kawasaki disease
MIS-C	multisystem inflammatory syndrome
NT-pro-BNP	N-terminal pro b-type natriuretic peptide
PLT	platelets
RT- PCR	real-time reverse-transcriptase–polymerase-chain-reaction
WHO	World Health Organization
